# Just the Facts: Assessing and managing soft tissue knee injuries in the Emergency Department

**DOI:** 10.1007/s43678-024-00761-w

**Published:** 2024-08-10

**Authors:** Benjamin Gompels, Luke McCarron, Luka Jovanovic, Thomas Molloy, Vazeer Ahmed, Martin Gargan, Mike Barrett

**Affiliations:** 1 Division of Trauma and Orthopaedics, Addenbrooke’s Hospital, Cambridge, UK; 2 Accident and Emergency Department, Addenbrooke’s Hospital, Cambridge, UK; 3 Bristol Children’s Hospital, Bristol, UK

## Case description

A 24-year-old man attends the with acute knee pain and swelling following a collision at a football match earlier in the day.

### What are the key clinical details in the history?

i. Mechanism of injury

Take a thorough history of the trauma that precipitated the injury. Clarify the position of the knee and the body at the time of injury, the direction of the force applied, and determine the timing of the incident. If swelling was immediately evident following a knee twisting motion, it could indicate an anterior cruciate ligament (ACL) injury or a fracture. In contrast, delayed swelling over 24 h can suggest a meniscal injury.

ii. Onset and location of pain

The nature, severity, location, duration, and onset of pain provide insight into the structures that may have been damaged [1]. Determining whether the pain was present immediately or developed following exercise is essential. Does climbing stairs elicit pain? This may indicate involvement of the patellofemoral joint. In addition, it is essential to inquire about how the pain impacts the patient’s daily activities and identify any exacerbating or relieving factors.

iii. Associated symptoms

The presence of associated symptoms and sounds experienced during the injury is crucial to note. Common symptoms include ‘shifting,’ ‘locking,’ ‘popping,’ and ‘sliding’ of the knee joint [1]. Establishing whether there is a feeling of instability or ‘giving way’ is also helpful. This can be broadly categorized into true stability, instability during daily activities, or pain-mediated instability [2]. Does the patient’s knee lock? This sensation may suggest a meniscal injury or a loose body. Differentiating between the sensation of the knee being locked and the knee being truly locked in a fixed position during the examination is useful.

iv. Past medical history

Is there any history of knee injuries or surgeries? If so, are there any residual functional deficits from these incidents?

### How do you examine this patient?

Following the history, both knees should be examined, the neurovascular status of the lower limb should be evaluated, and examinations should be conducted on the hip and ankle joints adjacent to the affected knee. The knee examination’s structure may follow the LOOK—FEEL—MOVE format**.**

- **LOOK**: observe for erythema, swelling, bruising, deformities, lacerations, or previous surgical incisions. Assess if the patient is weight-bearing—an inability to bear weight following the injury increases the likelihood of a fracture or high-grade ligament injury.
- **FEEL**: assess temperature, joint line and point tenderness, joint effusion and tendon defects
- **MOVE**: passive and active movement of the joint

Conclude with appropriate special tests (see Box 1).

Box 1: Special tests to identify the damaged structure in soft tissue knee injuries. The special tests are selected based on which structure is most likely affected by the pathology.

**Table:** 

Anterior cruciate ligament	Lachman’s test—highest sensitivity (86%) and specificity (91%) for diagnosing an ACL injury in an acute setting [3] Anterior drawer test—low sensitivity (20%) and high specificity (88%) [4] Lever test—high sensitivity (92.5%) and low specificity (25%) [3]
Posterior cruciate ligament	Posterior drawer test—high sensitivity (90%) and specificity (99%) [5] Posterior sag test—high sensitivity (90%) and specificity (100%) [5]
Medial collateral ligament	Valgus stress test—high sensitivity (91%) and low specificity (49%) when laxity is the outcome measure [6]
Lateral collateral ligament	Varus stress test—low sensitivity (25%) and specificity not reported [7]
Meniscal injury	No single test has been found to accurately diagnose meniscal tears Combination of tests, e.g., McMurray’s test, Apley’s test. Thessaly test is recommended
Quadriceps/patellar tendon	Straight leg raise

Box 2: Differential diagnoses in a traumatic knee injury

**Table:** 

Soft tissue differentials	Non-soft tissue differentials
Anterior/Posterior cruciate ligament (ACL/PCL) injury	Patellar fracture or dislocation, in particular patellofemoral dislocation
Medial/Lateral collateral ligament (MCL/LCL) injury	Tibial plateau fracture
Meniscus injury	Avulsion fracture
Patella tendon injury	Bone contusions
Quadriceps tendon injury	Tug lesions
Distal hamstring injury (biceps femoris, semitendinosus, semimembranosus)

### What investigations should I offer?

*Radiography*: Following trauma to the knee, the Ottawa Knee Rules state that a radiograph should be requested only for patients who meet at least one of the following:

- Age > / = 55
- Isolated tenderness of the patella
- Tenderness at the head of the fibula
- Inability to flex the knee to 90 degrees
- Inability to bear weight immediately and in the Emergency Department for four steps [2]

Radiographs can assess for fractures and other non-soft tissue differentials mentioned in Box 2, including osteochondroma and tug lesions.

*Ultrasonography*: *U*ltrasound imaging is a safe and cost-effective approach for confirming a joint effusion and evaluating superficial structures [8]. However, ultrasonography was inferior to MRI when diagnosing ruptures in the superficial structures of the quadriceps and patellar tendons [8]. That said, US imaging may be useful in assessing the patellar and quadriceps tendon in operators with sufficient skill where there is a delay in accessing MRI [9].

*Magnetic Resonance Imaging (MRI) and Computed Tomography (CT)*: MRI is a precise method that provides details of the knee’s soft tissues. It can be used to confirm the diagnosis of a soft tissue injury. CT may be used where a fracture is suspected despite normal knee radiography or more information about a previously confirmed fracture is required. CT is used primarily to assess tibial plateau fractures. MRI and CT may be most efficacious during follow-up of the initial injury.

### What are the management options in the acute setting?

Management involves taking a comprehensive history, conducting a detailed examination, and obtaining plain radiographs. MRI and US should be considered for diagnosing soft tissue injuries.

Updated UK NICE guidelines suggest adults should undergo immediate specialist assessment in the following situations:

- Evidence of neurovascular compromise—the neurovascular status can be determined by palpating the dorsalis pedis, posterior tibial and popliteal pulses and assessing the sensory and motor function of the peroneal and tibial nerve
- Soft tissue injury with significant gross instability can be challenging to assess in the acutely injured knee; consider arranging a follow-up for a repeat examination and any further testing or referrals [10].

Anterior cruciate ligament: Surgical treatment is recommended in young and athletic patients. Some patients will opt for conservative management. Following this, there will be a period of protected weight-bearing in a hinged brace, with the range of movement gradually increasing.

Posterior cruciate ligament: Initially, the aims are to reduce swelling, maintain range of movement and quadriceps activation. Indications for operative treatment include avulsion fractures, multi-ligamentous knee injuries, or failed conservative management.

Lateral and medial collateral ligament injury: Using crutches and a knee brace with knee locked in full extension for 4–6 weeks with weight-bearing as tolerated. Surgical reconstruction may be required for high-grade injuries.

Generally, the PRICER principles of Protection, Rest, Ice, Compression, Elevation, and Rehabilitation should be followed in managing soft tissue knee injuries. Except in specific cases like severe ligamentous injuries, total immobilization is generally avoided or minimized to maintain function and enhance recovery. Consequently, physiotherapy is often an essential part of the rehabilitation process. Patients should be provided with appropriate analgesia, and non-steroidal anti-inflammatory drugs (NSAIDs) can help reduce acute inflammation.

## Summary

- Soft tissue knee injuries commonly cause Emergency Department presentations and timely diagnosis promotes recovery.
- PRICER principles are effective in the majority of soft tissue knee injuries. More severe ligamentous injuries require specialist assessment.
- Follow-up is integral to the continuity of care for these injuries.
